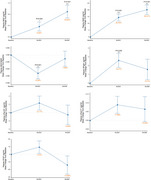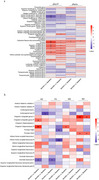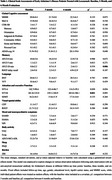# Effects of Lecanemab in Chinese Patients with Early Alzheimer's Disease: Evidence from a Multidimensional Real‐World Study

**DOI:** 10.1002/alz70861_108201

**Published:** 2025-12-23

**Authors:** Wenyan Kang, Chao Gao, Xiaoyan Li, Peijian Huang, Jun Liu

**Affiliations:** ^1^ Department of Neurology, Institute of Neurology, Ruijin Hospital, Shanghai Jiao Tong University School of Medicine, Shanghai, Shanghai China; ^2^ Ruijin Hospital, Shanghai Jiao Tong University School of Medicine, Shanghai China; ^3^ Department of Neurology, Hainan Branch, Ruijin Hospital, Shanghai Jiao Tong University School of Medicine, Shanghai, Shanghai China; ^4^ Department of Neurology, Institute of Neurology, Ruijin Hospital, Shanghai Jiao Tong University School of Medicine, Shanghai, Shanghai China; ^5^ Department of Neurology and Institute of Neurology, Ruijin Hospital affiliated to the Shanghai Jiaotong University School of Medicine, Shanghai, Shanghai China

## Abstract

**Background:**

Lecanemab has shown promise in treating early Alzheimer’s disease (AD), but its efficacy and safety in Chinese populations remain unexplored. Our study aims to provide the first comprehensive evaluation of lecanemab's efficacy and safety in a Chinese population within a real‐world setting, to investigate the effects of lecanemab on cognition, Aβ‐PET, plasma biomarkers, brain functional connectivity, and white matter integrity.

**Methods:**

This real‐world study evaluated the efficacy and safety of lecanemab in Chinese patients diagnosed with mild cognitive impairment (MCI) and mild AD. Evaluations included cognitive tests, plasma biomarker analysis, amyloid‐β PET, and advanced neuroimaging (fMRI, DTI). Safety was closely monitored, particularly for amyloid‐related imaging abnormalities (ARIA).

**Results:**

First, lecanemab demonstrated a favorable safety profile in Chinese patients. The incidence of adverse reactions, particularly ARIA, was significantly lower than reported in other studies. Second, cognitive stability was maintained after 6 months of treatment, with marked improvements in long‐delay recall, indicating lecanemab's potential to delay cognitive decline in Chinese patients effectively. Third, progressive increases in plasma Aβ42 and Aβ40 levels at 3 and 6 months post‐treatment, as well as a significant reduction in cerebral Aβ deposits shown by Aβ‐PET, both demonstrate lecanemab’s effective Aβ clearance. Changes in plasma neurofilament light chain (NFL) levels also suggest the neuroprotective potential of lecanemab. Additionally, fMRI and DTI analyses indicated that lecanemab might modulate brain functional connectivity and white matter integrity, potentially contributing to cognitive preservation.

**Conclusions:**

These findings highlight the safety and therapeutic potential of lecanemab in Chinese patients with early AD. Further studies with larger cohorts and longer follow‐up are needed to elucidate its mechanisms and disease‐modifying effects.